# Neural oscillations during acupuncture imagery partially parallel that of real needling

**DOI:** 10.3389/fnins.2023.1123466

**Published:** 2023-04-06

**Authors:** Hao Zhang, Qingxia Liu, Menglin Yao, Zhiling Zhang, Xiu Chen, Hua Luo, Lili Ruan, Tianpeng Liu, Yingshuang Chen, Jianghai Ruan

**Affiliations:** ^1^Department of Neurology, The Affiliated Hospital of Southwest Medical University, Luzhou, China; ^2^Laboratory of Neurological Diseases and Brain Function, Luzhou, China; ^3^School of Integrated Traditional Chinese and Western Medicine, Southwest Medical University, Luzhou, China; ^4^Shaanxi University of Chinese Medicine, Xianyang, China

**Keywords:** acupuncture imagery, microstates, KI3 (Taixi), needling, EEG

## Abstract

**Introduction:**

Tasks involving mental practice, relying on the cognitive rehearsal of physical motors or other activities, have been reported to have similar patterns of brain activity to overt execution. In this study, we introduced a novel imagination task called, acupuncture imagery and aimed to investigate the neural oscillations during acupuncture imagery.

**Methods:**

Healthy volunteers were guided to watch a video of real needling in the left and right KI3 (Taixi point). The subjects were then asked to perform tasks to keep their thoughts in three 1-min states alternately: resting state, needling imagery left KI3, and needling imagery right KI3. Another group experienced real needling in the right KI3. A 31-channel-electroencephalography was synchronously recorded for each subject. Microstate analyses were performed to depict the brain dynamics during these tasks.

**Results:**

Compared to the resting state, both acupuncture needling imagination and real needling in KI3 could introduce significant changes in neural dynamic oscillations. Moreover, the parameters involving microstate A of needling imagery in the right KI3 showed similar changes as real needling in the right KI3.

**Discussion:**

These results confirm that needling imagination and real needling have similar brain activation patterns. Needling imagery may change brain network activity and play a role in neural regulation. Further studies are needed to explore the effects of acupuncture imagery and the potential application of acupuncture imagery in disease recovery.

## 1. Introduction

Traditional acupuncture has been applied in China for thousands of years and has rapidly gained popularity as an alternative and complementary medicine owing to its therapeutic effects (Liang and Wu, 2006). Taixi (KI3), a Traditional Chinese Medicine (TCM) concept and a key acupoint of the kidney meridian, has been used to treat patients with cognitive impairment (Chen et al., 2014). Previous studies have confirmed that notion that acupoint stimulation can induce changes in brain functional connectivity (FC) (Chavez et al., 2017). For example, needling in KI3 could induce changes in FC involving sensory/somatomotor and subcortical networks (He et al., 2020); acupuncture treatment in patients with Mild Cognitive Impairment can increase FC between the left dorsal lateral prefrontal cortex (DLPFC) and left precuneus and decrease FC between the left DLPFC and left inferior temporal gyrus (Zhang et al., 2022). Especially, the effects of acupuncture on FC may indicate changes in clinical traits (Liu et al., 2022).

Electroencephalography (EEG) microstate (Ms) analysis, a widely accepted whole-brain imaging technique, is a reliable and reproducible EEG signal analysis technique that can depict the organization and temporal dynamics of large-scale brain cortical oscillations with high temporal resolution (Michel and Koenig, 2018). The rest-state EEG activity can be interpreted as a limited number of distinct quasi-stable states and can record the scalp’s topographies of electric potentials in a multichannel array, which remain stable for tens of milliseconds to more than 100 milliseconds (Khanna et al., 2015). Recent studies have classified microstates into four categories: microstates A, B, C, and D, which are associated with the temporal lobe, occipital cortex, partial cingulate, insula, frontal cortices, and frontal and parietal cortices, respectively (Michel and Koenig, 2018). All four microstates represent different functions involving auditory, speech, vision, significance, and dorsal attention networks (Britz et al., 2010). In summary, microstates can simultaneously analyze all electrode signals to create a global representation of a functional state (Serrano et al., 2018). Accumulating research has identified the Ms dynamic changes under migraine, mental tasks, drug intervention, etc., (Kim et al., 2021; Li et al., 2022; Ricci et al., 2022).

Tasks involving mental practice, such as thinking motors, have been reported to have similar patterns of brain activity to overt execution (Kraeutner et al., 2014). Interestingly, this kind of mental stimulation can alter brain connectivity and may help in the recovery of stroke patients, although controversy remains (Dickstein et al., 2013; Hatem et al., 2016; Silva et al., 2020; De Filippi et al., 2022). Therefore, it is reasonable to expect that the stimulation and execution of other tasks will have both similar and differentiated brain activity patterns.

In this study, we introduced a novel concept called acupuncture imagery. We hypothesized that mental practice in acupuncture and real acupuncture execution show some common and different brain activities. For that purpose, those subjects were introduced to remain in a resting state, needling imagery in left KI3, needling imagery in right KI3, or received real needling in right KI3 and synchronous EEG was recorded. Brain activity patterns were investigated by using EEG microstates.

## 2. Materials and methods

### 2.1. Participants

A total of 50 right-handedness young college students who had experienced real acupuncture (major TCM) and provided written informed consent were recruited from the Affiliated Hospital of Southwest Medical University. Thirty subjects were randomly divided into the acupuncture imagery (AI) task group and 20 subjects were randomly divided into the real acupuncture (RA) needling group. Here we using the function *randperm* in Matlab to produce the random number sequences. None of the included subjects reported any history of neurological or psychiatric disorders. The EEG of all the subjects showed a normal rhythm. This study was approved by the Ethics Committee of the Affiliated Hospital of Southwest Medical University (KY2019008).

### 2.2. Data acquisition

#### 2.2.1. Preparation before collection

Before data collection, participants in the AI group were instructed to watch a pre-recorded real acupuncture paradigm video (3 min for one acupoint). The video shows an experienced acupuncturist needling at the left KI3 and right KI3 acupoints of the model. The needles were inserted perpendicularly to a depth of approximately 2 cm at KI3, and the inserted needle was manipulated by rotating the needle clockwise and counterclockwise 360° in each rotation and lifting-thrusting. The acupuncturist performed each rotating and lifting thrusting alternately for 30s. Two needle manipulations were performed for 3 min in at each acupoint. Volunteers in both the AI and RA groups were informed of the research protocol.

#### 2.2.2. Task protocol

**Figure 1:**
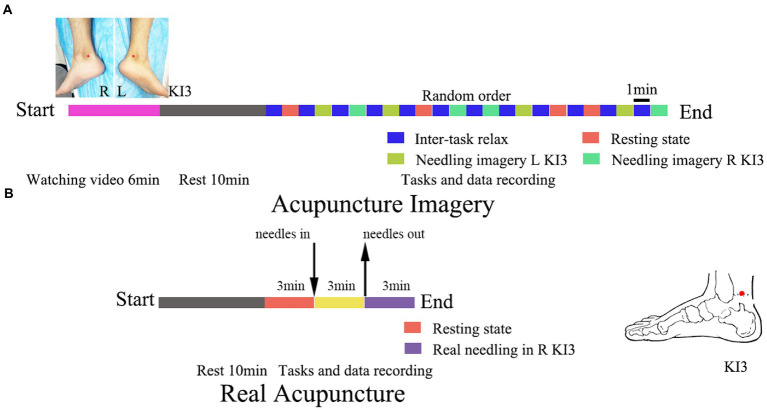
Trial flowchart of acupuncture imagery **(A)** and real acupuncture **(B)**. The location of acupoint KI3 has been marked with red dot. L, left; R, right.

The volunteers in the AI group were requested to perform three 1-min tasks: keeping thoughts wandering in the resting state, needling imagery in the left KI3, and needling imagery in the right KI3. The three tasks were executed four times in a random alternation sequence and a one-minute relax epoch was performed between each task (Figure 1).

The volunteers in the RA group were introduced to remain in a rest state for 3 min, and then an experienced acupuncturist executed the acupuncture operations for 3 min. The location placement of KI3 and the needling method were in accordance with the video used in AI group and our previous study (He et al., 2020). Disposable sterile acupuncture needles (0.25 mm*30 mm, Zhongyan, Beijing, China) were used for acupuncture. After needling out, the EEG was recorded for further 3 min (Figure 1).

All the processes were introduced using an E-Prime (v2.0) program (Psychology Software Tools, Inc. United States) through a screen in front of the participants and the participants sat in an enclosed silent room during the trials.

#### 2.2.3. EEG collection

For all subjects, during the tasks, EEG was synchronously recorded using an analog recorder (Brain Products, Germany) and the electrodes were placed using a 31-channel brain cap. The impedance of the electrodes was maintained at <5 kΩ. The EEG markers were input using the E-Prime software (v2.0, Psychology Software Tools, Inc. United States). The sample rate was 1,000 Hz for all recordings. After data collection, EEG epochs in the resting state, needling imagery in the left KI3, and needling imagery in the right KI3 were extracted. The EEG data in resting state and post needling in right KI3 were cut for 4 min for each condition of each subject in the AI group. Three minutes were required for each condition for each subject in the RA group.

### 2.3. EEG preprocessing

The EEG preprocessing steps were similar to those described in a previous study (Zhou et al., 2021). Briefly, the EEG was bandpass filtered between 1 Hz and 45 Hz using a Hamming window after the FIR filter. Then, electromyogram artefacts and muscle artifacts were completely removed using the EEGlab plugin-in AAR.^1^ In this tool, the blind source separation algorithm was employed to perform electrooculogram (fast ICA algorithm) corrections automatically, and the EEG matrix with a low fractal dimension was identified as electrooculogram components and was removed automatically (Seitzman et al., 2017). The same algorithm was used to correct the electromyogram automatically (Serrano et al., 2018). Finally, the EEG data for each subject were re-computed to the average reference, and the sample rate was resampled to 200 Hz.

After preprocessing, the EEG of interest was extracted and saved in text format for subsequent Ms analysis. In the AI group, we extracted 4 min for each condition. For the RA group, we extracted 2 min for each condition.

### 2.4. Microstate analysis

EEG microstate analysis was performed using the LORETA-Key tool (v20190617, Bain-heart: KEY Institute in Zurich, Switzerland).^2^ The detailed method used in this study is described in a previous study (Liu et al., 2021). In brief, the extracted EEG data were used to calculate the global field power (GFP) of EEG signals at each time point using Eq. (1):


(1) $$GFP\left(t\right)=\sqrt{\frac{\sum_{i=1}^{n}{\left[\text{vi}\left(t\right)-v\left(t\right)\right]}^{2}}{n}}$$

*n* is the number of electrodes, vi (*t*) represents the potential value of the i-th electrode at time *t*, v(*t*) represents the average potential of all electrodes at a time, and GFP reflects the degree to which the potential between the electrodes changes at a given time. The local maximum of the GFP curve has the strongest signal strength and highest signal-to-noise ratio, and the potential distribution at the local maximum of the GFP curve maintains a stable state. Therefore, the topographic map at the maximum point of GFP was used to represent the topographic map around it for analysis. Subsequently, k-means clustering analysis (ignoring spatial polarity) was conducted to identify the most important original topographic map, and the original topographic map was divided into several types. In this study, a cluster number ranging from 4 to 8 were tested with all the EEG data. The optimal number of clusters was determined *via* cross-validation. The number of clusters with the lowest cross-validation (CV) value was recognized and extracted as subject-representative microstate topographies. The topographical map of the scalp potential of each subject at each time point was compared with the microstates obtained by clustering, and the original topographic map was matched with the label corresponding to the microstates according to the correlation size and marked A-D.

After extracting the individual’s Ms, the corresponding time-series parameters of the EEG Ms were calculated. Average duration: average length of time for a given Ms to remain stable when it occurs; frequency of occurrence: average number of occurrences of the microstate per second; percentage of time/coverage of microstates: percentage of specified microstates in the total recorded time; Markov chain transition probability between different microstates: the probability of changing from the current Ms to another state.

### 2.5. Statistical analysis

One-way ANOVA was conducted for the three subgroup comparisons within the AI group and *post-hoc* Tukey–Kramer tests were performed for pairwise comparisons. For the RA group, paired *t*-test was used to compare the microstate parameters from the two conditions, and comparisons between the AI and RA groups in the microstate parameters were assessed using a two-sample *t*-test after a generalized linear model (GLM) with age, sex and resting state microstate parameters regressed out. Cohen’s *d* was computed for each comparison to assess the effect size. Multiple comparisons were corrected with the FDR method at level *p* < 0.05. Only FDR-adjusted *p* < 0.05 was considered significant. All tests were performed using MATLAB (R2014a, MathWorks Inc.).

## 3. Results

### 3.1. Demographic details

Thirty (aged 21.7 ± 1.12 years) and 20 (aged 21.5 ± 2.4 years) healthy participants were included in AI and RA group, respectively. No significant differences in age or sex between the two groups were found (Table 1).

**Table 1:** Demographics of subjects included in the study.

	AI group (*n* = 30)	RA group (*n* = 20)	*χ2/t*	*p*
Male:female	11:19	8:12	0.057	0.812^#^
Age (years, mean ± SD)	21.7 ± 1.12	21.5 ± 2.40	−0.398	0.692^##^

AI, acupuncture imagery; RA, real acupuncture.

# Chi-square test.

## Two sample *t*-test.

### 3.2. Microstates analysis

According to the cross-validation analysis, the cluster number ‘4’ showed the lowest CV errors (Supplementary Table S1). Thus, cluster number ‘4’ was the best fit for the present data. Based on the spatial orientation of these maps, we labelled the four microstate maps as A (left–right orientation), B (right–left orientation), C (anterior–posterior orientation) and D (fronto-central maximum) for these conditions, as previous studies (Michel and Koenig, 2018; Liu et al., 2021; Zhou et al., 2023). We found significant differences in the parameters within the AI group or within the RA group and between the AI and RA groups.

#### 3.2.1. Comparisons within AI group

**Figure 2:**
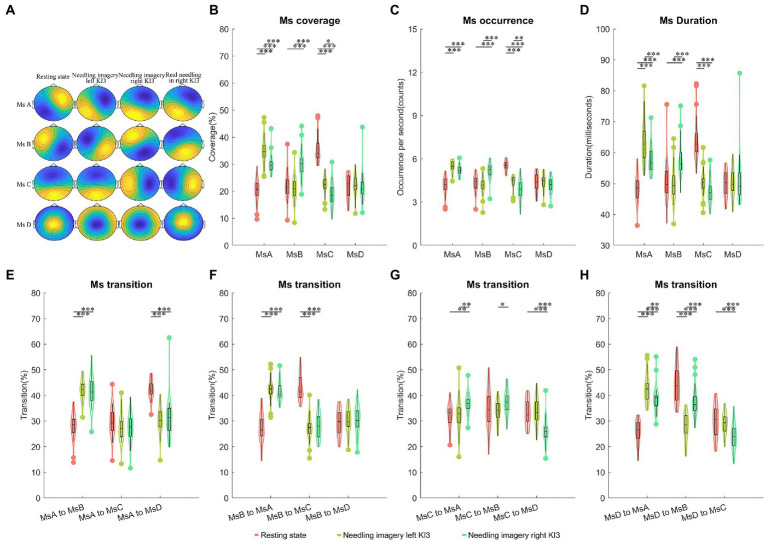
Temporal dynamics of EEG microstates within AI group. **(A)** The four microstates were recognized by k-means cluster analysis across subjects in the four conditions (resting state, needling imagery in left KI3, needling imagery right KI3 within AI group, and real needling in right KI3 of RA group). **(B)** The coverage of the four microstates in each condition within AI group. **(C)** The occurrence per second of the four microstates in each condition within AI group. **(D)** The duration of the four microstates in each condition within AI group. **(E–H)** Markov chain transition probabilities of the four microstates in each condition within AI group. *Post-hoc* Tukey–Kramer tests were carried out when the ANOVA revealed significant group differences at *p* < 0.05. *post hoc* test with FDR correction ^*^p < 0.05, ^**^p < 0.01, ^***^p < 0.001; Ms, microstate; AI, acupuncture imagery.

Compared with needling imagery in KI3, the EEG from the resting state showed the lowest time coverage for microstate A (MsA) (*post hoc p* < 0.05) but showed the highest coverage for MsC (*post-hoc p* < 0.05). The resting state showed decreased coverage in microstate B (MsB), compared to needling imagery in the right KI3 (*post-hoc p* < 0.05). These trends were reserved for other Ms parameters, including the occurrence and duration of MsA, MsB and microstate (MsC) (*post-hoc p* < 0.05). The microstate D (MsD) of the three Ms parameters was not significant under the three conditions. In addition, compared with imaging, the Ms transitions between MsA and MsB decreased significantly in the resting state. The transition from MsA to MsD was upgraded, from MsD to MsA; However, it was downgraded in resting state (*post-hoc p* < 0.05). The transition probabilities from MsD to MsB and MsB to MsC decreased in the resting state (*post-hoc p* < 0.05) (Figure 2; Table 2; Supplementary Tables S2, S3).

**Table 2:** Summary of changes of microstate parameters in acupuncture imagery and real needling in KI3.^*^

Microstate parameters	Needling imagery in left KI3^#^	Needling imagery in right KI3^#^	Real needling in right KI3^#^	Needling imagery in right KI3^##^
**Microstate A**				
Coverage	↗	↗	↗	↗
Occurrence	↗	↗	↗	—
Duration	↗	↗	↗	↗
Transition A to B	↗	↗	—	↗
Transition A to C	—	—	↗	↘
Transition A to D	↘	↘	↘	—
**Microstate B**				
Coverage	—	↗	↘	—
Occurrence	—	↗	↘	—
Duration	—	↗	↘	↗
Transition B to A	↗	↗	↗	—
Transition B to C	↘	↘	↘	↘
Transition B to D	—	—	—	↗
**Microstate C**				
Coverage	↘	↘	↗	—
Occurrence	↘	↘	↗	—
Duration	↘	↘	↗	—
Transition C to A	—	↗	↗	↗
Transition C to B	—	—	↘	—
Transition C to D	—	↘	—	↘
**Microstate D**				
Coverage	—	—	↘	—
Occurrence	—	—	↘	—
Duration	—	—	↘	—
Transition D to A	↗	↗	—	—
Transition D to B	↘	↘	↗	—
Transition D to C	—	↘	↘	—

The up or down arrows indicate significantly increased or decreased results of the corresponding parameters in the corresponding comparisons (*p* < 0.05).

# Compared with the resting state.

## Compared with real needling in the right KI3.

Moreover, dynamic brain differences between needling imagery in left KI3 and the right KI3 were found. The MsA and MsC parameters, including coverage, occurrence, and duration, decreased significantly when the task was from needling imagery in the left KI3 to the right KI3 (*post-hoc p* < 0.05). However, these parameters for MsB showed an increasing trend when they experienced the same task switching (*post-hoc p* < 0.05). For those parameters involving Ms transition, the transitions from MsC to MsA or MsB, and from MsD to MsB increased significantly during thinking needling in the right KI3 (*post-hoc p* < 0.05). However, the transitions from MsD to MsA or MsC and from MsC to MsD decreased significantly during thinking needling in the right KI3 (*post-hoc p* < 0.05) (Figure 2; Supplementary Tables S2, S3).

#### 3.2.2. Comparisons within RA group

**Figure 3:**
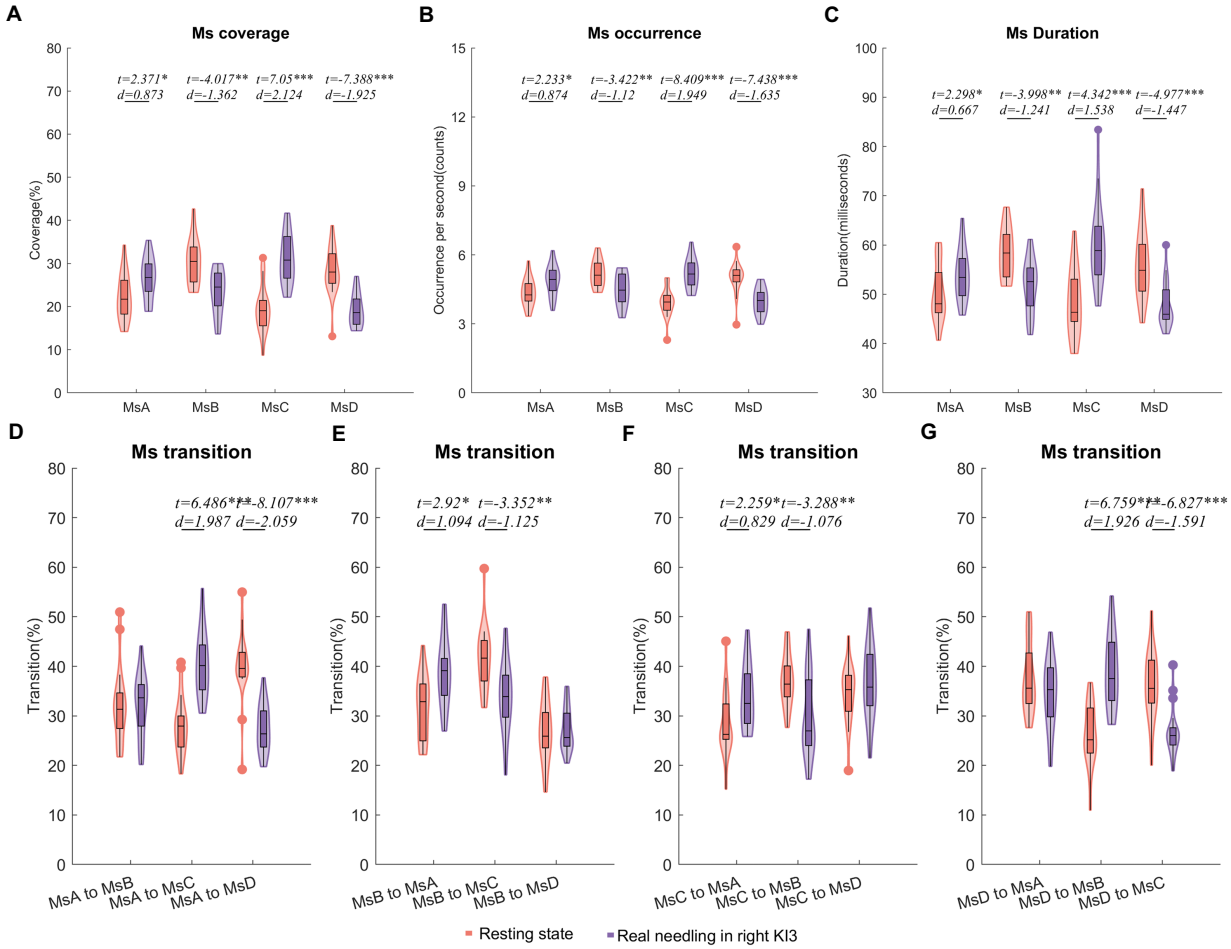
Temporal dynamics of EEG microstates in RA group. **(A)** The coverage of the four microstates in each condition within RA group. **(B)** The occurrence per second of the four microstates in RA group. **(C)** The duration of the four microstates in RA group. **(D–G)** Markov chain transition probabilities of the four microstates in RA group. Paired *t*-test with FDR correction ^*^p < 0.05, ^**^p < 0.01, ^***^p < 0.001; The *d* values indicated the effect size (Cohen’s *d*). Ms, microstate; RA, real acupuncture.

Compared with the resting state, real needling in right KI3 showed significantly increased coverage in MsA and MsC but decreased coverage in MsB and MsD (FDR *p* < 0.05). Moreover, the other Ms parameters, including the onset of Ms and duration involving the same Ms, showed the same trends (Figure 3; Table 2; Supplementary Table S4).

Furthermore, the Ms transitions between MsA and MsC increased significantly (FDR *p* < 0.05), but Ms transitions between MsB and MsC decreased significantly in EEG from real needling in the right KI3 (FDR *p* < 0.05), compared with the resting state EEG. The transition probabilities from MsA to MsD and from MsD to MsC were significantly lower in real acupuncture at the right KI3 than in the resting state. In contrast, the transition ratios from MsD to MsB and from MsB to MsA were higher in real acupuncture conditions than in the resting state (Figure 3; Table 2; Supplementary Table S4).

#### 3.2.3. Comparisons between needling imagery and real needling in right KI3

**Figure 4:**
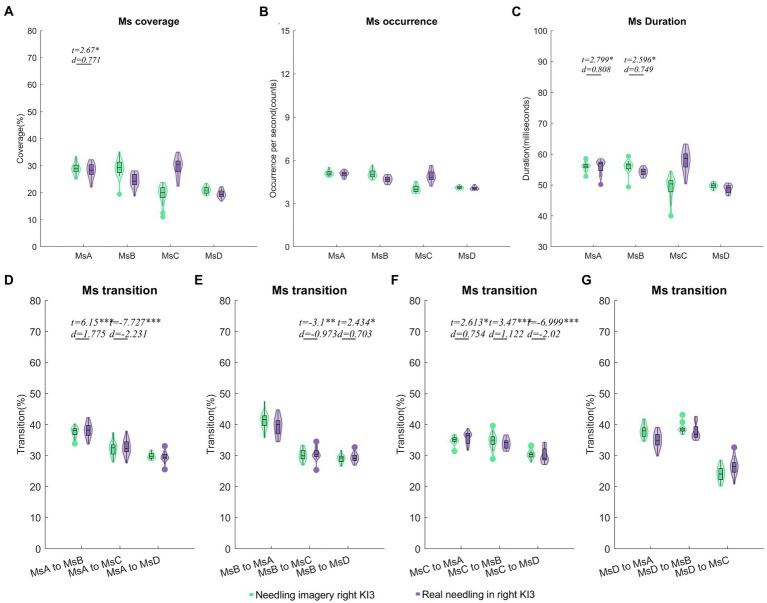
Comparisons of temporal dynamics between needling imagery and real needling in right KI3. Comparison results of coverage **(A)**, occurrence per second **(B)**, duration **(C)** and Markov chain transition probabilities **(D–G)** of the four microstates. In these comparisons, a two-sample *t* test after a generalized linear model (GLM) with age, sex and resting state microstate parameters was regressed out. Two sample *t* test with FDR correction ^*^p < 0.05, ^**^p < 0.01, ^***^p < 0.001; *d* values indicate effect size (Cohen’s *d*). Ms, microstate; RA, real acupuncture.

After controlling for age and sex, we found that the coverage of MsA and the duration of MsA and MsB were upgraded in needling imagery in KI3 when compared with real needling in the right KI3 (FDR *p* < 0.05). For the parameters of Ms transitions, the transitions from MsA to MsB, from MsB to MsD and from MsC to MsA or MsB were more likely to occur in the EEG from thinking needling in the right KI3 than that from real needling in the right KI3. Additionally, the relative transition probability from MsA or MsB to MsC and MsC to MsD decreased in needling imagery conditions, compared with real needling (Figure 4; Table 2; Supplementary Table S5).

## 4. Discussion

Few studies have focused on imagining acupuncture. In the present study, we applied EEG microstate analysis to detect alterations in neural oscillations during tasks involving acupuncture imagery and real needling. We recognized that brain activity changed in both the needling imagery and needling tasks. Interestingly, the parameters involving MsA of needling imagery in the right KI3 showed similar changes to real needling in the right KI3. The combined findings provide evidence of brain functional oscillations in real and simulated acupuncture at the electrophysiological imaging level. These findings confirm that needling imagination and real needling have parallel brain activation patterns.

Thinking needling and real needling showed similar microstate dynamics: increased coverage ratio, occurrence, mean duration of MsA, the transition from MsB or MsC to MsA, decreased transition from MsA to MsD, and from MsB or MsD to MsC. These findings indicate that real needling and needling imagery share common brain oscillations involving MsA. Considering that MsA has been reported to represent functional networks involving auditory (Michel and Koenig, 2018) and one important function of KI3 according to TCM theory is related to auditory (Zhu et al., 2015), we assumed that the parallel brain activity changes involving MsA suggested that both real and needling imagery in KI3 may play a role in functions involving auditory processing. Fewer microstate parameters were found to show differences between needling imagery and real needling in KI3 when we directly compared the two conditions, which further supported our assumption that acupuncture imagery is partially parallel to real needling.

However, some microstate parameters, such as MsB duration in needling imagery and real needling in the right KI3, showed significant differences and opposite trends compared with their resting state EEG. A previous study (Seitzman et al., 2017) found that MsB represented a visual network; the increased MsB duration in needling imagery and decreased MsB duration in real needling may indicate that the visual networks were more frequently used in tasks involving needling imagery.

In addition, MsC is recognized as a portion of the default mode network (DMN) (Michel and Koenig, 2018). Compared with the resting state, the MsC parameters including coverage, occurrence, and duration in the AI group decreased and in the RA group increased, which was not unexpected considering that the DMN was a task-negative network and the activity of the DMN decreased during the performance of cognitive tasks (Anticevic et al., 2012). Therefore, the upgraded and downgraded MsC parameters including coverage, occurrence, and duration during real needling and needling imagery may suggest that cognitive processes are more involved in real needling than acupuncture thinking tasks.

Decreased MsD parameters including coverage, occurrence, and duration in real needling compared to the resting state were demonstrated in the present study, which was not found in needling thinking tasks. Previous studies have confirmed that MsD is associated with the dorsal attention network (DAN) (Britz et al., 2010). Therefore, it is reasonable to speculate that DAN is inhibited during real acupuncture manipulation, as evidenced by the decreased MsD.

Overall, compared with needling imagery in the left KI3, the parameters of MsB including coverage, occurrence, and duration were increased in needling imagery in the right KI3, while other parameters such as the coverage ratios of MsA and MsB, the occurrence of MsC, and the duration of MsA decreased significantly. Interestingly, these trends between thinking left and right are in good agreement with a similar but more studied task: motor imagery, which has been studied using EEG microstates in previous reports and found similar differences between thinking left and right motor (Liu et al., 2017). These common findings may be caused by the functional asymmetry of the brain, where the spatial skills are mainly dependent on right hemisphere processing (Witelson, 1988; Ocklenburg and Gunturkun, 2012). We speculated that thinking tasks involving space on the right side could evoke more visual activity in the brain. More studies with paradigm tasks are needed to address this issue, despite one study finding the biased effects of tasks on the processing of visual information in two hemispheres (Naert et al., 2018).

Recently, several studies have found the motor mental practice can provide several benefits in patients with neurological disorders (Kim and Lee, 2015; Gil-Bermejo-Bernardez-Zerpa et al., 2021). Changes in the functional network induced by motor imagery may contribute to the recovery from brain dysfunction (Bajaj et al., 2015; Yu et al., 2022). Especially, imaging motors have been reported to have similar patterns of brain activity to real execution (Kraeutner et al., 2014). Our study found that acupuncture imagery and real needling in the right KI3 had the same neural network oscillations, especially networks involving MsA. Therefore, it is reasonable to assume that needling imagery may change brain network activity to play a role in neural regulation or can be coupled with real needling to enhance the therapeutic effect of acupuncture.

In terms of methodology, the Ms analysis used in this study is one method of spatial domain analysis. Compared to the classical frequency or time domain analysis, Not only does the Ms make full use of the high temporal resolution of EEG, but also it considers the spatial effect of EEG. Other methods such as scalp electrode PLV-based (Zhou et al., 2021) or source ROI-based FC analysis (Xie et al., 2022) often need prerequisites. The EEG Ms sequences exhibit scale-free dynamics and have a clearly structured temporal organization that is neither random nor predetermined. One of the disadvantages may be the low spatial resolutions of the source localization of microstates, which may be the common disadvantage of scalp EEG techniques.

The limitations of this study include a relatively small sample size. We did not address the longitudinal effects of needling imagery on microstates. Another limitation of the study is that the participants in this experiment were all healthy people experienced acupuncture (TCM students). In future studies, we will continuously collect more and broader populations to overcome these limitations. Patients will be included to detect the effects of acupuncture imagery on brain activity in the pathological state.

## 5. Conclusion

Using this EEG approach based on microstate analysis, we were able to demonstrate that neural oscillations changed in both needling imagery and real needling tasks. Some parameters, especially MsA, of needling imagery in the right KI3 showed similar changes to real needling in the right KI3. The combined findings provide evidence of the brain functional oscillations in real and simulated acupuncture at the electrophysiological imaging level. Needling imagery may change brain network activity and play a role in neural regulation. These findings support the notion that needling imagination and real needling have parallel brain activation patterns.

## Data availability statement

The raw data supporting the conclusions of this article will be made available by the authors, without undue reservation.

## Ethics statement

The studies involving human participants were reviewed and approved by the Ethics Committee of the Affiliated Hospital of Southwest Medical University (KY2019008). The patients/participants provided their written informed consent to participate in this study.

## Author contributions

JR: conceptualization, supervision, formal analysis, investigation, and review and editing of the manuscript. XC and HL: supervision, formal analysis, and review and editing of the manuscript. HZ, QL, MY, and ZZ: investigation, writing the original draft of the manuscript. HZ and QL: formal analysis and investigation. LR, TL, and YC: investigation and data curation. All authors contributed to the article and approved the submitted version.

## Funding

This work was supported by the Youth Program of the National Natural Science Foundation of China (81804198), Strategic Cooperation Project between Sichuan University and Luzhou Government (2021CDLZ-10), National Innovation and Entrepreneurship Training Projects for College Students (202010632075), and Innovation and Entrepreneurship Training Projects of Southwest Medical University (2020598).

## Conflict of interest

The authors declare that the research was conducted in the absence of any commercial or financial relationships that could be construed as a potential conflict of interest.

## Publisher’s note

All claims expressed in this article are solely those of the authors and do not necessarily represent those of their affiliated organizations, or those of the publisher, the editors and the reviewers. Any product that may be evaluated in this article, or claim that may be made by its manufacturer, is not guaranteed or endorsed by the publisher.

## Footnotes

^1^ http://germangh.com

^2^ www.uzh.ch/keyinst/loreta
