# Supplementary material for: Neural oscillations during acupuncture imagery partially parallel that of real needling
Source: Front Neurosci. 2023 Apr 6;17:1123466. doi: 10.3389/fnins.2023.1123466 (PMC10115979; doi:10.3389/fnins.2023.1123466)
Supplement: Supplementary file 1 [file Data_Sheet_1.docx]

S.Table 1 the cross-validation analysis of all the input EEG data.

| No. of Ms | CV errors |
| --- | --- |
| **4** | **0.028** |
| 5 | 0.029 |
| 6 | 0.032 |
| 7 | 0.034 |
| 8 | 0.037 |

S.Table 2 One-way ANOVA tests of Ms parameters between the resting-state, imaging needling in left KI3 and in right KI3.

| Microstate parameters | F | P |
| --- | --- | --- |
| **Coverage** |  |  |
| Ms A | 89.67 | <0.001 |
| Ms B | 31.69 | <0.001 |
| Ms C | 121.30 | <0.001 |
| Ms D | 0.27 | 0.761 |
| **Occurrence** |  |  |
| Ms A | 71.39 | <0.001 |
| Ms B | 28.81 | <0.001 |
| Ms C | 76.04 | <0.001 |
| Ms D | 1.32 | 0.274 |
| **Duration** |  |  |
| Ms A | 62.67 | <0.001 |
| Ms B | 15.85 | <0.001 |
| Ms C | 85.69 | <0.001 |
| Ms D | 0.09 | 0.914 |
| **Transition Probability** |  |  |
| MsA to MsB | 70.67 | <0.001 |
| MsA to MsC | 1.78 | 0.175 |
| MsA to MsD | 42.19 | <0.001 |
| MsB to MsA | 88.68 | <0.001 |
| MsB to MsC | 101.55 | <0.001 |
| MsB to MsD | 0.22 | 0.805 |
| MsC to MsA | 6.89 | 0.002 |
| MsC to MsB | 3.92 | 0.024 |
| MsC to MsD | 24.21 | <0.001 |
| MsD to MsA | 87.26 | <0.001 |
| MsD to MsB | 55.20 | <0.001 |
| MsD to MsC | 11.79 | <0.001 |

S.Table 3 The results of post hoc analysis using Tukey-Kramer after ANOVA tests between the resting-state, imaging needling in left KI3 and in right KI3^#^.

^#^The paired-comparisons were performed using post hoc Tukey-Kramer tests. ^##^These corresponding parameters showed no significant differences in the ANOVA tests were not taken into post hoc analysis. Ms, Microstate.

| Microstate parameters | Resting-state vs. imaging needling in left KI3 | | Resting-state vs. imaging needling in right KI3 | | Imaging needling in left KI3 vs. right KI3 | |
| --- | --- | --- | --- | --- | --- | --- |
|  | Difference | *P* | Difference | *P* | Difference | *P* |
| **Coverage** |  |  |  |  |  |  |
| Ms A | -14.636 | <0.001 | -9.703 | <0.001 | 4.934 | <0.001 |
| Ms B | 1.178 | 0.617 | -7.989 | <0.001 | -9.167 | <0.001 |
| Ms C | 13.811 | <0.001 | 17.161 | <0.001 | 3.350 | 0.014 |
| Ms D^##^ | — | — | — | — | — | — |
| **Occurrence** |  |  |  |  |  |  |
| Ms A | -1.354 | <0.001 | -1.079 | <0.001 | 0.276 | 0.061 |
| Ms B | 0.156 | 0.540 | -0.880 | <0.001 | -1.037 | <0.001 |
| Ms C | 1.164 | <0.001 | 1.639 | <0.001 | 0.476 | 0.002 |
| Ms D^##^ | — | — | — | — | — | — |
| **Duration** |  |  |  |  |  |  |
| Ms A | -14.808 | <0.001 | -8.808 | <0.001 | 6.000 | <0.001 |
| Ms B | 0.872 | 0.837 | -6.992 | <0.001 | -7.864 | <0.001 |
| Ms C | 14.600 | <0.001 | 17.176 | <0.001 | 2.576 | 0.169 |
| Ms D^##^ | — | — | — | — | — | — |
| **Transition Probability** |  |  |  |  |  |  |
| MsA to MsB | -14.296 | <0.001 | -13.611 | <0.001 | 0.686 | 0.869 |
| MsA to MsC | — | — | — | — | — | — |
| MsA to MsD | 12.047 | <0.001 | 11.204 | <0.001 | -0.843 | 0.833 |
| MsB to MsA | -14.914 | <0.001 | -14.024 | <0.001 | 0.890 | 0.759 |
| MsB to MsC | 15.536 | <0.001 | 14.849 | <0.001 | -0.686 | 0.843 |
| MsB to MsD^##^ | — | — | — | — | — | — |
| MsC to MsA | -0.029 | 1.000 | -4.419 | 0.005 | -4.390 | 0.005 |
| MsC to MsB | 0.664 | 0.883 | -2.999 | 0.086 | -3.663 | 0.027 |
| MsC to MsD | -0.635 | 0.875 | 7.418 | <0.001 | 8.053 | <0.001 |
| MsD to MsA | -16.428 | <0.001 | -12.185 | <0.001 | 4.243 | 0.004 |
| MsD to MsB | 16.114 | <0.001 | 6.488 | <0.001 | -9.626 | <0.001 |
| MsD to MsC | 0.315 | 0.969 | 5.698 | <0.001 | 5.383 | <0.001 |

S.Table 4 The comparisons of Ms parameters between the resting-state and real needling in right KI3

| Microstate parameters | Resting-state  (mean±SD) | Real needling in right KI3  (mean±SD) | *P*^#^ | *t* | *d^##^* |
| --- | --- | --- | --- | --- | --- |
| **Coverage** |  |  |  |  |  |
| Ms A | 22.12±5.20 | 26.54±4.91 | 0.033 | 2.371 | 0.873 |
| Ms B | 30.36±5.18 | 23.40±5.04 | 0.001 | 4.017 | -1.362 |
| Ms C | 19.33±5.37 | 30.96±5.58 | <0.001 | 7.050 | 2.124 |
| Ms D | 28.18±5.48 | 19.10±3.80 | <0.001 | 7.388 | -1.925 |
| **Occurrence** |  |  |  |  |  |
| Ms A | 4.35±0.60 | 4.90±0.66 | 0.038 | 2.233 | 0.874 |
| Ms B | 5.20±0.56 | 4.49±0.69 | 0.004 | 3.422 | -1.120 |
| Ms C | 3.94±0.62 | 5.17±0.64 | <0.001 | 8.409 | 1.949 |
| Ms D | 5.00±0.68 | 3.99±0.56 | <0.001 | 7.438 | -1.635 |
| **Duration** |  |  |  |  |  |
| Ms A | 50.28±5.84 | 53.91±5.01 | 0.036 | 2.298 | 0.667 |
| Ms B | 58.16±5.03 | 51.70±5.38 | 0.001 | 3.998 | -1.241 |
| Ms C | 48.28±6.63 | 59.91±8.38 | 0.001 | 4.342 | 1.538 |
| Ms D | 56.07±6.74 | 47.68±4.69 | <0.001 | 4.977 | -1.447 |
| **Transition Probability** |  |  |  |  |  |
| MsA to MsB | 31.97±7.51 | 32.19±6.32 | <0.001 | 0.138 | 0.032 |
| MsA to MsC | 28.09±5.76 | 40.27±6.49 | <0.001 | 6.486 | 1.987 |
| MsA to MsD | 39.95±7.07 | 27.54±4.77 | <0.001 | 8.107 | -2.059 |
| MsB to MsA | 31.62±6.24 | 38.78±6.84 | 0.011 | 2.920 | 1.094 |
| MsB to MsC | 41.51±6.44 | 33.87±7.14 | 0.004 | 3.352 | -1.125 |
| MsB to MsD | 26.87±6.08 | 27.36±4.46 | <0.001 | 0.353 | 0.091 |
| MsC to MsA | 28.31±6.57 | 33.62±6.24 | 0.037 | 2.259 | 0.829 |
| MsC to MsB | 37.16±5.21 | 29.76±8.22 | 0.005 | 3.288 | -1.076 |
| MsC to MsD | 34.54±5.84 | 36.62±7.20 | <0.001 | 0.994 | 0.318 |
| MsD to MsA | 37.63±7.14 | 34.37±6.70 | <0.001 | 1.845 | -0.472 |
| MsD to MsB | 26.21±6.17 | 38.91±6.99 | <0.001 | 6.759 | 1.926 |
| MsD to MsC | 36.16±6.78 | 26.72±4.93 | <0.001 | 6.827 | -1.591 |

^#^ P, Paired *t* test after FDR correction. ^##^cohen’s d.

S.Table 5 The comparisons of Ms parameters between needling imagery and real needling in right KI3.

| Microstate parameters | Needling imagery  (mean±SD) | Real needling in right KI3  (mean±SD) | *P*^#^ | *t* | *d^##^* |
| --- | --- | --- | --- | --- | --- |
| **Coverage** |  |  |  |  |  |
| Ms A | 1.10±3.20 | -1.64±4.04 | 0.014 | 2.670 | 0.771 |
| Ms B | 0.84±4.61 | -1.26±5.04 | 0.000 | 1.523 | 0.440 |
| Ms C | -0.82±3.87 | 1.23±6.04 | 0.000 | 1.344 | -0.423 |
| Ms D | 0.32±5.23 | -0.48±4.04 | 0.000 | 0.584 | 0.168 |
| **Occurrence** |  |  |  |  |  |
| Ms A | 0.10±0.37 | -0.15±0.56 | 0.000 | 1.734 | 0.542 |
| Ms B | 0.12±0.56 | -0.19±0.69 | 0.000 | 1.742 | 0.503 |
| Ms C | -0.17±0.73 | 0.25±0.73 | 0.000 | 1.997 | -0.577 |
| Ms D | 0.06±0.52 | -0.09±0.53 | 0.000 | 0.949 | 0.274 |
| **Duration** |  |  |  |  |  |
| Ms A | 1.38±4.10 | -2.06±4.49 | 0.012 | 2.799 | 0.808 |
| Ms B | 1.72±5.97 | -2.58±5.38 | 0.014 | 2.596 | 0.749 |
| Ms C | -1.39±4.56 | 2.09±8.16 | 0.000 | 1.737 | -0.558 |
| Ms D | 0.75±7.79 | -1.12±4.99 | 0.000 | 1.037 | 0.275 |
| **Transition Probability** |  |  |  |  |  |
| MsA to MsB | 4.02±5.89 | -6.03±5.29 | 0.000 | 6.150 | 1.775 |
| MsA to MsC | -5.07±5.30 | 7.60±6.22 | 0.000 | 7.727 | -2.231 |
| MsA to MsD | 1.33±7.71 | -1.99±4.17 | 0.000 | 1.964 | 0.507 |
| MsB to MsA | 0.44±3.33 | -0.66±5.98 | 0.000 | 0.748 | 0.241 |
| MsB to MsC | -2.15±4.44 | 3.22±6.85 | 0.003 | 3.100 | -0.973 |
| MsB to MsD | 1.35±5.13 | -2.03±4.29 | 0.019 | 2.434 | 0.703 |
| MsC to MsA | 1.44±4.21 | -2.16±5.51 | 0.014 | 2.613 | 0.754 |
| MsC to MsB | 2.77±4.38 | -4.15±8.17 | 0.001 | 3.470 | 1.122 |
| MsC to MsD | -4.40±4.77 | 6.60±6.34 | 0.000 | 6.999 | -2.020 |
| MsD to MsA | 0.42±5.03 | -0.63±6.26 | 0.000 | 0.660 | 0.190 |
| MsD to MsB | -0.79±5.93 | 1.18±6.23 | 0.000 | 1.129 | -0.326 |
| MsD to MsC | -0.23±4.61 | 0.34±4.13 | 0.000 | 0.446 | -0.129 |

^#^ *P*, two sample *t* test with resting-state regressed out after FDR correction. ^##^cohen’s d.
